# Minocycline binds and inhibits LYN activity to prevent STAT3-meditated metastasis of colorectal cancer

**DOI:** 10.7150/ijbs.70708

**Published:** 2022-03-21

**Authors:** Ling Yang, Jing Yang, Hua Liu, Jingbin Lan, Ying Xu, Xiaobo Wu, Yushan Mao, Dongming Wu, Kejian Pan, Tao Zhang

**Affiliations:** 1 School of Basic Medical science, Chengdu Medical College, Chengdu, China.; 2 School of Bioscience and Technology, Chengdu Medical College, Chengdu, China.; 3 The Second Affiliated Hospital of Chengdu Medical College, China National Nuclear Corporation 416 Hospital, Chengdu, China.; 4 School of Clinical Medicine, The First Affiliated Hospital of Chengdu Medical College, Chengdu, Sichuan 610500, P.R. China.

**Keywords:** Minocycline, LYN, STAT3, EMT, colorectal cancer

## Abstract

Colorectal cancer (CRC) is one of the most common malignancies worldwide. Metastasis is a major cause of CRC recurrence and mortality. Several antibiotic drugs have been reported to exert potential anticancer activities, however, whether and how the tetracycline antibiotic minocycline exhibit tumor suppressive effect on CRC remains unknown. Here, we found that minocycline markedly inhibits the epithelial-mesenchymal transition (EMT) process and metastasis of CRC cells both *in vitro* and *in vivo*. Using chemical proteomics screening combined with docking analysis and site-directed mutagenesis, we identified LYN as a direct bind target of minocycline, and Ala255 of LYN is required for minocycline binding. Mechanistically, minocycline binding inactivates LYN, leading to STAT3 inactivation and EMT suppression, thereby inhibits CRC metastasis. Tissue microarray analysis further confirmed the clinical relevance of LYN-STAT3 axis in the EMT and progression of CRC. In addition to CRC, minocycline also significantly prevents EMT process and inhibits the metastasis of several other cancer types. Our findings elucidate the mechanism of action of minocycline for the inhibition of CRC metastasis by LYN binding, and suggest that repurposing minocycline may represent a promising strategy for the treatment of advanced CRC and other cancer types.

## Introduction

Colorectal cancer (CRC) is the third most common malignancy and the second leading cause of cancer-related mortality worldwide 1, 2. Although the diagnostic and therapeutic efficacy has taken great strides during the past decades, the overall prognosis of CRC patients still remain poor 3, 4. Most CRC patients have poor prognosis due to metastasis and recurrence 5. Despite surgical treatment, radiotherapy and chemotherapy, the therapeutic effect is minimal for patients with advanced CRC. Therefore, it is imperative to develop effective therapeutic drugs for the treatment of metastatic CRC 6, 7.

The Src family of kinases were commonly overexpressed or aberrantly activated for the survival, growth, and metastasis of cancer cells 8, 9. Recently studies showed elevated levels of Src kinase LYN in colorectal 10, breast 11, prostate 12 and lung cancer cells 13. Notably, LYN could promote epithelial-mesenchymal transition (EMT) in breast and bladder cancers 14. In addition, LYN was reported to bind with, phosphorylate, and subsequently activate signal transducer and activator of transcription 3 (STAT3), leading to increased expression of EMT-inducing transcription factors to favor the metastasis of cervical cancer cells 15. These studies suggest that LYN is a promising therapeutic target for the treatment of metastatic cancer. However, to date LYN inhibitors have not been clinically used for cancer management. Hence, developing novel inhibitors of LYN is urgently required and will hold great promise for the therapy of advanced cancer.

There is increasing evidence that antibiotic drugs exhibit potential anticancer effects, such as doxorubicin 16, mitoxantrone 17, bleomycin 18 and mitomycin-C 19. Minocycline, a second-generation tetracycline derivative, has been found to exert antioxidant, anti-inflammatory, neuroprotective effects, in addition to its antibacterial effects 20, 21. Recent studies have reported that minocycline inhibits the growth of glioma cells, providing the possibility of using minocycline for the cancer treatment 22, 23. However, whether minocycline has inhibitory effect on CRC remains to be determined. More importantly, the molecular target of minocycline underlying its anticancer activity is unclear.

In this study, we have identified LYN as a direct binding target of minocycline. Minocycline binding inhibits the kinase activity of LYN, leading to STAT3 inactivation, EMT suppression and metastatic prevention in CRC cells. The anti-metastatic effect is also elucidated in several other cancer types. Our data unravel the mechanism of action of minocycline regarding its anticancer effect, and provide a rationale for the use of minocycline for advanced cancer treatment.

## Results

### Minocycline prevents CRC metastasis by inhibiting EMT *in vitro* and *in vivo*

**Figure 1:**
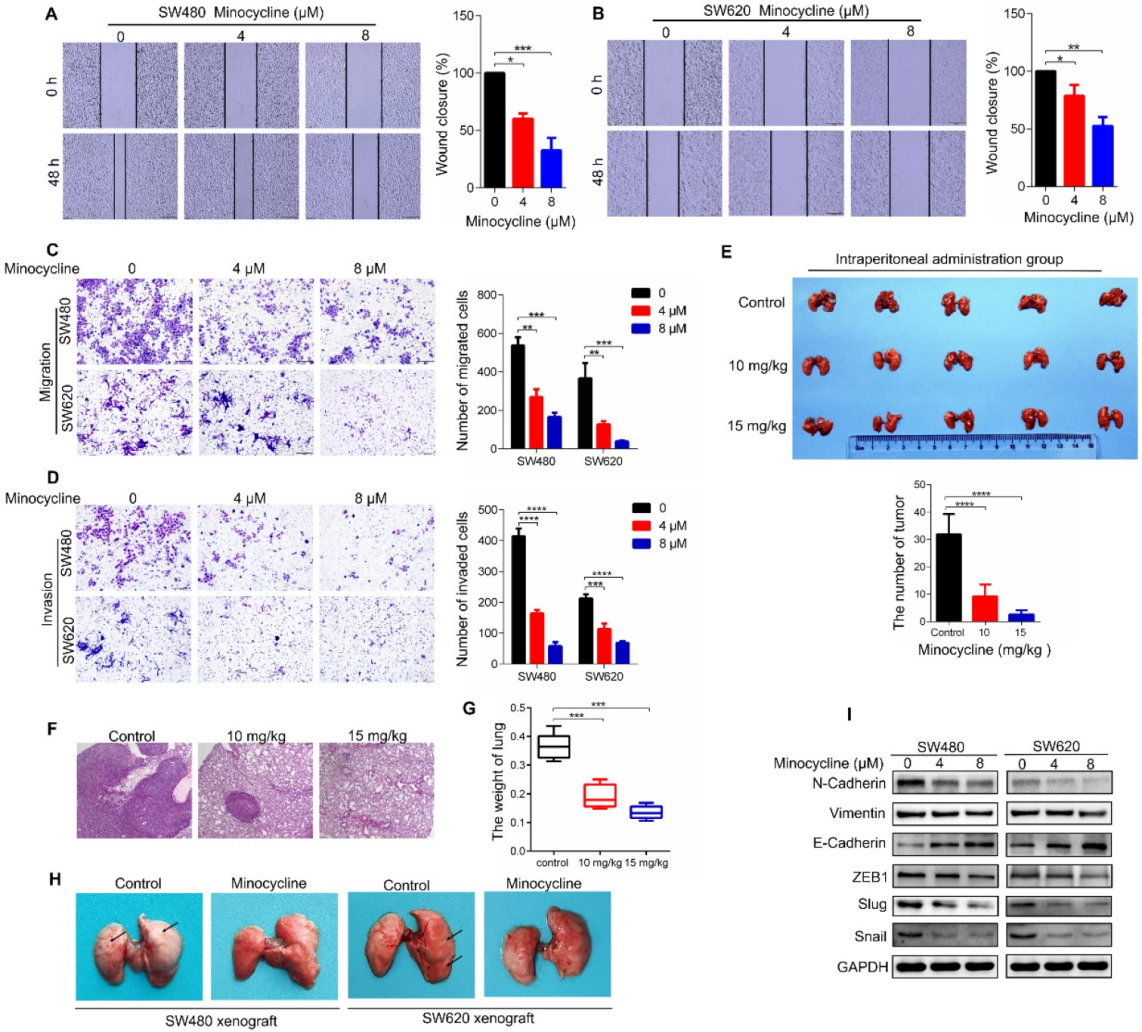
** Minocycline prevents CRC metastasis by inhibiting EMT *in vitro* and *in vivo*. (A-B)** Wound-healing migration assay for SW480 (A) and SW620 (B) cells treated with minocycline with indicated concentrations. The wound space was photographed at 0 and 48 h. Representative images of wounds and the statistical results at each concentration were recorded. **(C-D)** Representative images for transwell migration (C) and Matrigel invasion (D) assays for indicated colorectal cancer cells treated with minocycline. **(E)** Representative images and statistical analysis of the number of metastatic nodules in the lungs of mice following intraperitoneal injection of minocycline. **(F-G)** H&E staining of mice lung tissues and the lung weight (G) following intraperitoneal injection of minocycline. **(H)** Representative images of mice lung metastasis of SW480 and SW620 cells following intraperitoneal injection of minocycline. **(I)** The levels of EMT markers following minocycline treatment detected by western blot analysis. Error bars indicate the mean ± SD of three independent experiments. Scale bar, 200 µm.^*^, P < 0.05; ^**^, P < 0.01; ^***^, P < 0.001; ^****^, P < 0.0001.

To investigate whether the antibiotic drug minocycline exhibits anticancer effect against CRC cells, we first examined the effect of minocycline on the growth of CRC cells. Using MTT and colony formation assay, we found that minocycline inhibited the growth of CRC cells at high concentrations (> 8 μM), but has no obvious effect on CRC cell growth at relatively low concentrations (0-8 μM) (Figure S1A-C). Surprisingly, minocycline treatment at either 4 μM or 8 μM significantly suppressed the mobility capacity of CRC cells as evidenced by wound-healing migration assay in a dose-dependent manner (Figure 1A-B). Transwell migration and matrigel invasion assays further confirmed that minocycline effectively inhibited the migration and invasion of SW480 and SW620 cells Figure 1C-D). These results suggest that minocycline treatment inhibits the migration and invasion of CRC cells at low concentrations (4 μM and 8 μM), but has no obvious effect on the cell growth at the same doses.

To determine whether minocycline represses the metastasis of CRC cells *in vivo*, a lung metastasis model was generated by injecting CT26 mouse CRC cells into the tail of BALB/c mice. As shown in Figure 1E-F, minocycline-treated mice showed a smaller number of lung metastatic nodules compared with the control group. In addition, minocycline treatment markedly reduced the weight of lung in mice (Figure 1G). The decreased lung metastatic nodules and lung weight were further confirmed in another lung metastasis model, in which minocycline treatment was conducted in cultured CT26 cells before injection (Figure S2A-C). Consistently, minocycline also had an inhibitory effect on the lung metastasis of tail-injected SW480 or SW620 human CRC cells (Figure 1H). Together, these data indicate that minocycline significantly inhibits the metastasis of CRC cell *in vivo*.

It is well known that EMT plays an important role in cancer metastasis. We thus examined whether minocycline suppresses EMT process in CRC cells by detecting the protein levels of several EMT markers. Compared with the control group, the expression levels of Vimentin and N-Cadherin were suppressed in minocycline-treated SW480 and SW620 cells, while the level of E-Cadherin was elevated (Figure 1I). Moreover, minocycline treatment resulted in significant reduction in the levels of EMT-associated transcription factors, including Snail, Slug and ZEB1 (Figure 1I). Overall, these results suggest that minocycline suppresses CRC metastasis by inhibiting EMT process.

### Minocycline directly binds and inhibits LYN activity

**Figure 2:**
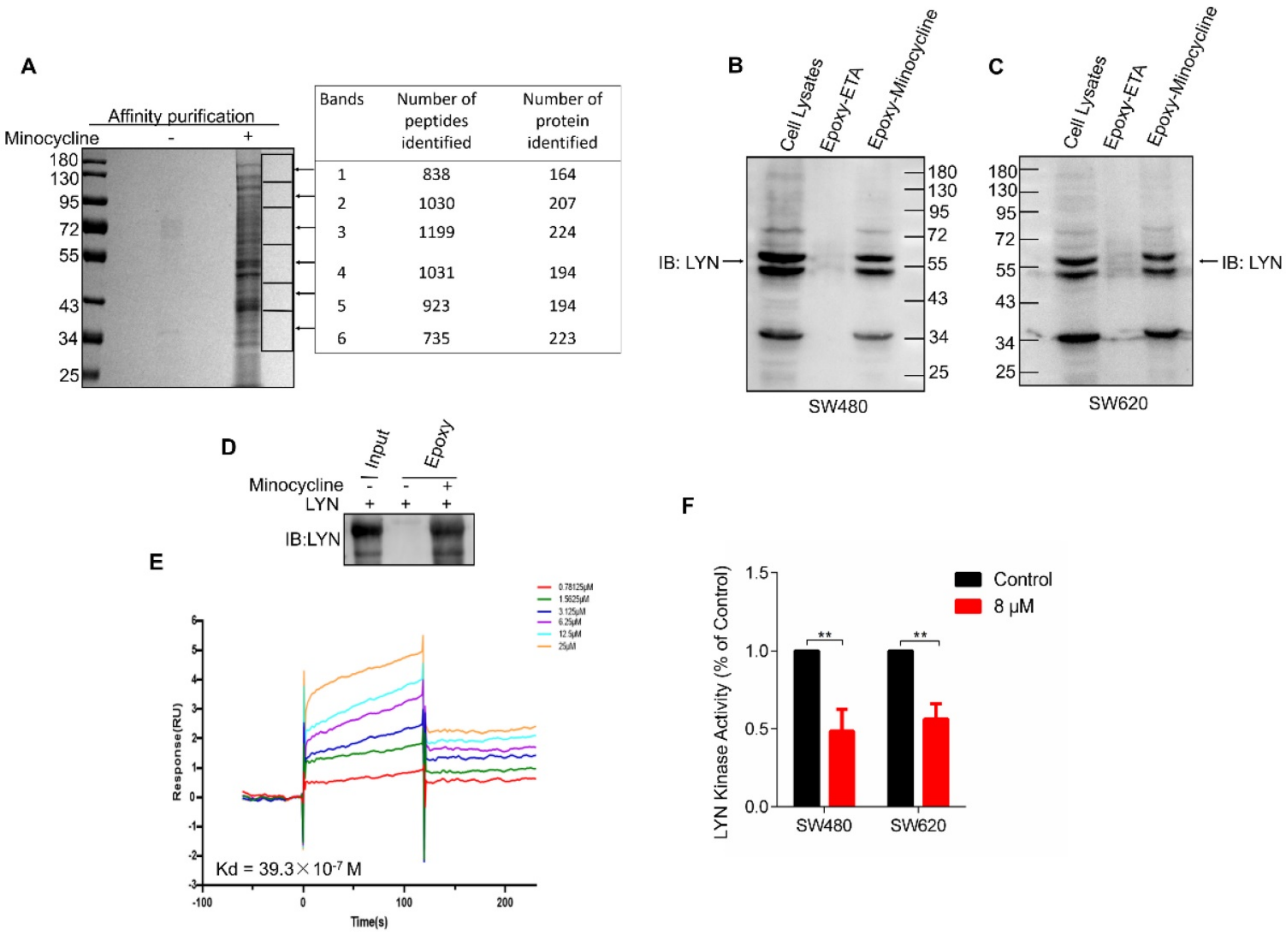
** Minocycline directly binds and inhibits LYN activity. (A)** Affinity purification of minocycline binding protein from SW480 cell lysates. Epoxy-actived μSphere beads were fixed with minocycline, incubated with SW480 cell lysates and washed to remove non-specifical bound contaminants. The SDS-PAGE gel was stained with Coomassie, and candidate binding targets were listed (right). **(B-C)** Minocycline was immobilized to Epoxy-actived μSphere beads, incubated with SW480 or SW620 cell lysates, and the binding proteins were subjected to pull down and western blot analysis with anti-LYN antibody. **(D)** Recombinant human LYN was incubated with minocycline-immobilized beads for affinity purification and then immunoblotted with anti-LYN antibody. **(E)** Surface plasmon resonance (SPR) analysis the interaction of minocycline with increasing concentrations of recombinant human LYN. **(F)** The kinase activity of LYN in SW480 or SW620 cells were determined by tyrosine kinase assay kit. Error bars indicate the mean ± SD of three independent experiments. ^**^, P < 0.01.

In order to investigate the mechanism of action of minocycline's anti-CRC effect, chemical proteomics approach was performed to identify the cellular binding target of minocycline. Minocycline was coupled to Epoxy-activated μSphere beads and then incubated with SW480 cell lysates. The minocycline-binding proteins were affinity-purified, subjected to SDS-PAGE and then analyzed by mass spectrometry (MS). A series of proteins potentially binding with minocycline were identified (Figure 2A). Increasing evidence showed that the Lyn tyrosine kinase, a member of the Src family of kinases (SFKs), can promote EMT 24-26. Meanwhile, there is evidence that Lyn regulation of VAV1 in cancers activates the Rac1-PAK1 kinase cascade to stabilize Slug and Snail, allowing them to initiate transcription of EMT genes 14, so the metastasis-related tyrosine protein kinase LYN attracted our attention. The binding of minocycline with LYN in SW480 and SW620 cell lysates was then confirmed by affinity purification coupled with western blot analysis (Figure 2B-C). To determine whether this binding is direct, we purified recombinant human LYN protein and found that recombinant LYN protein can also be affinity purified by minocycline (Figure 2D). Surface plasmon resonance (SPR) analysis further confirmed this direct interaction (Figure 2E). Overall, these findings indicate that the tyrosine protein kinase LYN is a direct binding target of minocycline. Meanwhile, we examined LYN activity and found that minocycline treatment significantly inhibited the LYN enzyme activity in SW480 and SW620 cells (Figure 2F). In summary, these data indicate that minocycline directly binds and inhibits LYN activity.

### Ala255 of LYN is required for minocycline binding

**Figure 3:**
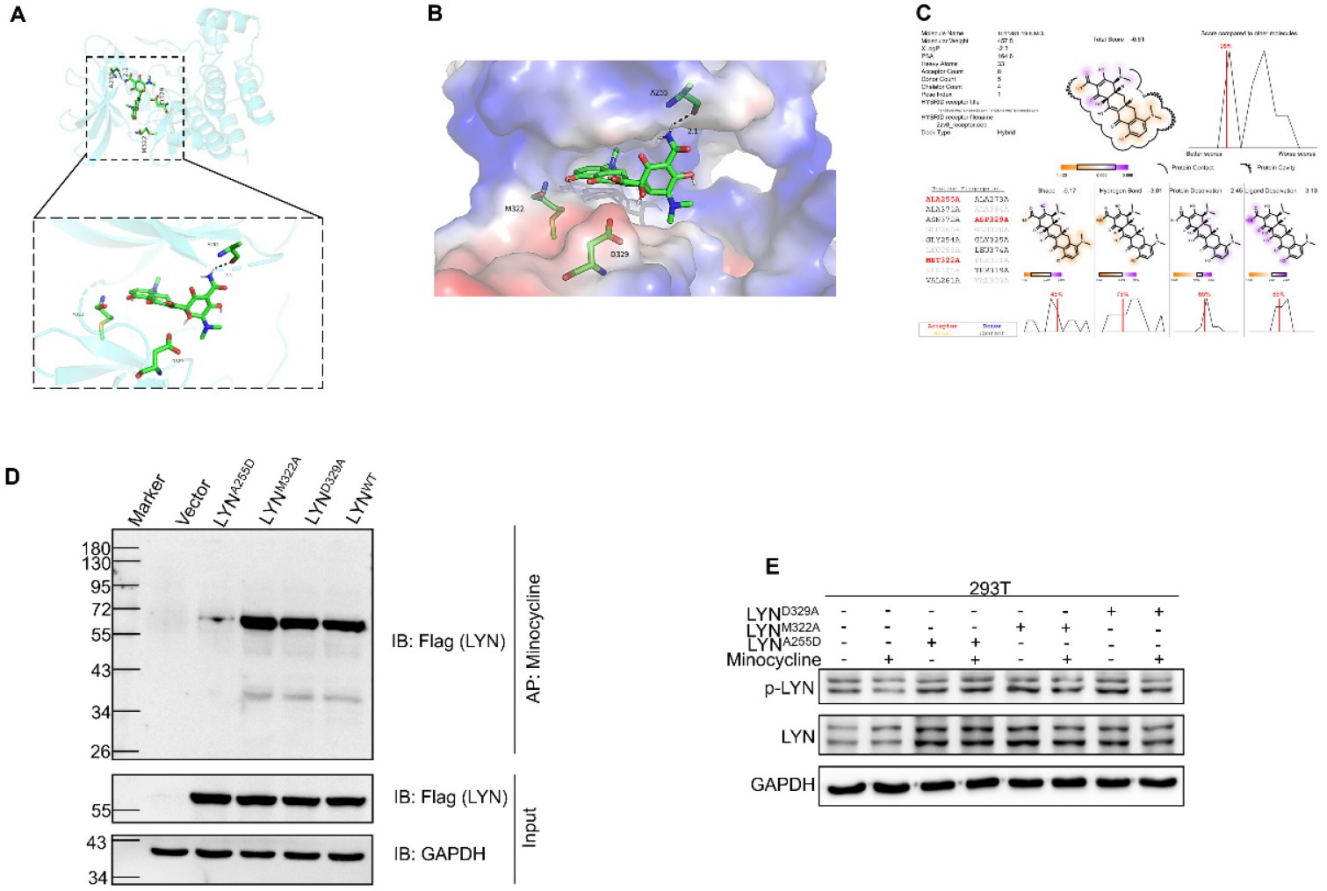
** Ala255 of LYN is required for minocycline binding. (A-B)** Cartoon representation (A) and electrostatic surface representation (B) of the docking model of minocycline binding to *H. sapiens* LYN. **(C)** The possible binding sites of LYN to minocycline were determined by docking analysis. **(D)** Following transfection of LYN^WT^ or LYN mutant plasmids in 293T cells, the Flag-LYN^WT^ and LYN mutant proteins were affinity-purified with Epoxy-actived μSphere immobilized with minocycline, and then immunoblot analysis with anti-Flag antibody. **(E)** Following transfection of LYN mutant plasmids in 293T cells with or without minocycline treatment, western blot analysis was performed with antibodies against p-LYN and LYN.

We next set out to determine the binding site of LYN with minocycline. Using docking analysis, three possible binding sites, Ala255 (A255), Met322 (M322) and Asp329 (D329), were identified (Figure 3A-C). We then constructed point-mutant plasmids of the three amino acids (A255D, M322A, D329A) to determine which amino acid is required for minocycline binding. Compared with the wild-type (LYN^WT^) group, LYN^A255D^mutant has almost no interaction with minocycline, whereas both LYN^M322A^and LYN^D329A^ mutants retain minocycline binding activity (Figure 3D). In addition, minocycline significantly decreased the phosphorylation level of LYN^M322A^ or LYN^D329A^ mutants, but not LYN^A255D^ mutant (Figure 3E). These data suggest that Ala255 of LYN is required for minocycline binding.

### Minocycline inhibits the migration and invasion of CRC cells by directly binding LYN

**Figure 4:**
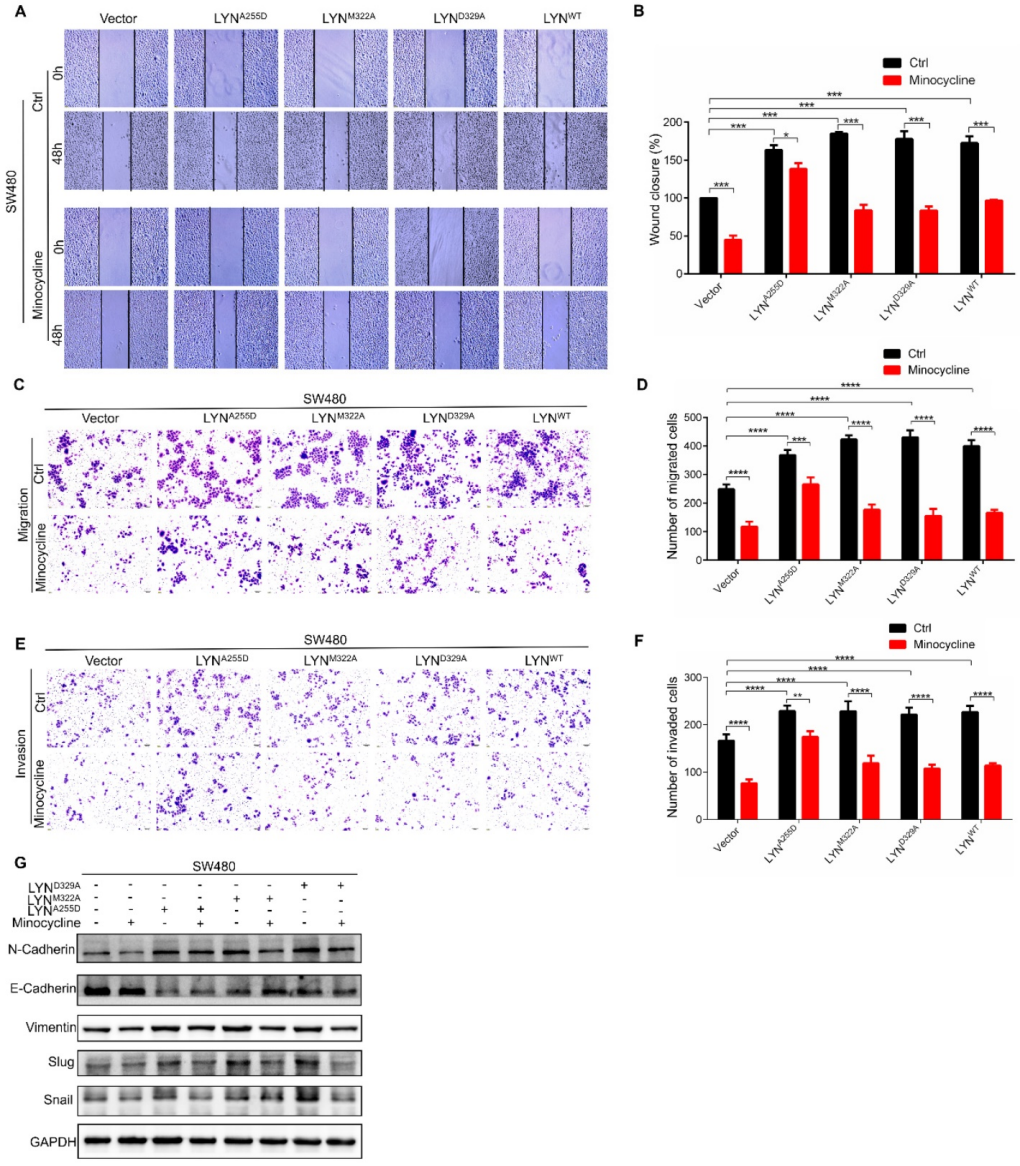
** Minocycline inhibits the migration and invasion of CRC cells by directly binding LYN. (A-B)** The wound healing migration analysis was performed in SW480 cells transfected with LYN^WT^ or LYN mutant plasmids followed by minocycline treatment. The wound space was photographed at 0 and 48 hours. Representative images of wounds and the statistic results were recorded. **(C-D)** Transwell migration assay was performed in SW480 cells transfected with LYN^WT^ or LYN mutant plasmids followed by minocycline treatment. **(E-F)** Matrigel invasion assay was performed in SW480 cells transfected with LYN^WT^ or LYN mutant plasmids followed by minocycline treatment. **(G)** The expression of EMT markers was detected by western blot analysis in SW480 cells transfected with LYN^WT^ or LYN mutant plasmids followed by minocycline treatment. Error bars indicate the mean ± SD of three independent experiments. Scale bar, 200 µm.^*^, P < 0.05; ^**^, P < 0.01; ^***^, P < 0.001; ^****^, P < 0.0001.

Given that minocycline suppresses CRC metastasis, and minocycline reduces LYN activity by direct binding at Ala255, we speculated that the metastasis inhibitory effect of minocycline may be attributed by LYN binding. To test this hypothesis, we overexpressed LYN^A255D^, LYN^M322A^ and LYN^D329A^ mutants and LYN^WT^ in SW480 cells, which exhibits relatively low basal LYN protein level. Using wound-healing migration assay, we found that enforced expression of either LYN^WT^, LYN^A255D^, LYN^M322A^ or LYN^D329A^ markedly promoted the wound closure rate. The increased scratch healing ability of LYN^WT^, LYN^M322A^ or LYN^D329A^-expressing cells was markedly reduced by minocycline. However, minocycline treatment had no significant effect on the mobility capacity of LYN^A255D^-expressing cells (Figure 4A-B). These observations were further recapitulated by transwell migration and matrigel invasion assays (Figure 4C-F). Moreover, we detected the expression levels of EMT-associated proteins, such as E-Cadherin, Vimentin, N-Cadherin, Snail and Slug after transfecting LYN^A255D^, LYN^M322A^, LYN^D329A^ and LYN^WT^ plasmids. As shown in Figure 4G, overexpressing LYN^WT^ or LYN mutants significantly reduced the E-Cadherin level and increased the levels of N-Cadherin, Vimentin, Slug and Snail. Notably, minocycline treatment obviously inhibited these changes, with the exclusion of LYN^A255D^-expressing cells. These results indicate that the binding of LYN is required for the anti-metastatic capacity of minocycline in CRC cells.

### Minocycline-LYN binding inhibits STAT3 signaling

**Figure 5:**
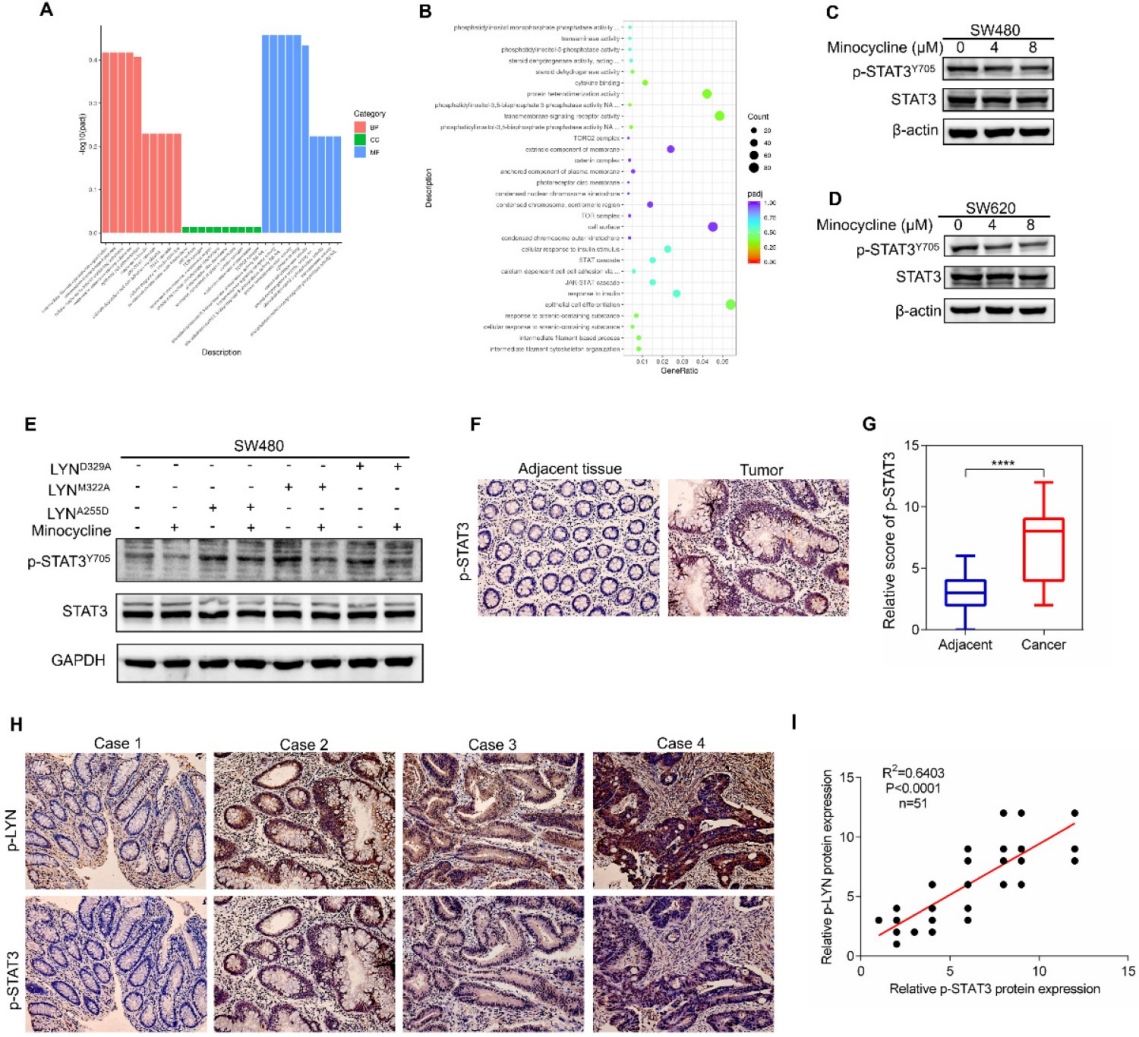
** Minocycline-LYN binding inhibits STAT3 signaling. (A-B)** STAT3 signaling pathway is suppressed by minocycline as shown by GO-Biological Process enrichment analysis. **(C-D)** SW480 or SW620 cells were treated with minocycline (0, 4, 8 µM) for 48 h, the protein levels of STAT3, p-STAT3^Y705^ were analyzed by western blot. **(E)** The expression of STAT3 and p-STAT3 in SW480 cells transfected with LYN^WT^ or LYN mutant plasmids followed by minocycline treatment. **(F)** Immunohistochemical staining was performed to detect the expression of p-STAT3 in the patient's CRC tissues and adjacent tissues. **(G)** The score of p-STAT3 staining in (F). **(H)** Representative images of immunohistochemical staining of p-LYN and p-STAT3 in CRC tissues. **(I)** The correlation of p-LYN and p-STAT3 levels in (H). ^****^, P < 0.0001.

To investigate the mechanism underlying minocycline-LYN binding-mediated CRC metastatic inhibition, we performed RNA sequencing (RNA-seq) to interrogate the changes of global gene expression in the absence and presence of minocycline. GO-Biological Process enrichment analysis showed that STAT cascade was suppressed by minocycline treatment (Figure 5A-B). Previous studies showed that LYN can bind with STAT3, leading to STAT3 activation 27, 28. In addition, it has been reported that STAT3 can promote metastasis by promoting the expression of EMT-related transcription factors 29, 30. In this regard, we presumed that minocycline-repressed CRC metastasis may be attributed to LYN binding and inactivation, and subsequent STAT3 inhibition. Indeed, minocycline significantly reduced the phosphorylation level of STAT3 at Tyr705 (Y705) (Figure 5C-D). We then overexpressed different LYN mutants in SW480 cells with or without minocycline treatment, and found that LYN mutant-overexpressing cells showed higher levels of phosphorylated STAT3 (Y705). Minocycline treatment markedly attenuated the increase of STAT3 phosphorylation in LYN^M322A^ and LYN^D329A^-overexpressing cells, but not in LYN^A255D^-overexpressing cells (Figure 5E). These results suggest that minocycline inhibits STAT3 by binding and inactivating LYN.

To evaluate the correlation of LYN and STAT3 in CRC clinical samples, we first determined the clinical relevance of p-STAT3 in 51 CRC tissues. As shown in Figure 5F-G, p-STAT3 staining in CRC tissues was more robust than that in adjacent normal tissues. More importantly, we found that the level of p-LYN is positively correlated with p-STAT3 level in CRC tissues (Figure 5H-I). Although we cannot directly evaluate the effect of minocycline-LYN binding in STAT3 inhibition and CRC metastasis suppression in CRC patients, these clinical data combined with our *in vitro* results support the notion that minocycline inhibits LYN-STAT3 signaling in CRC cells by direct LYN binding.

### Phosphorylated LYN level is elevated in CRC tissues and positively correlates with CRC metastasis

**Figure 6:**
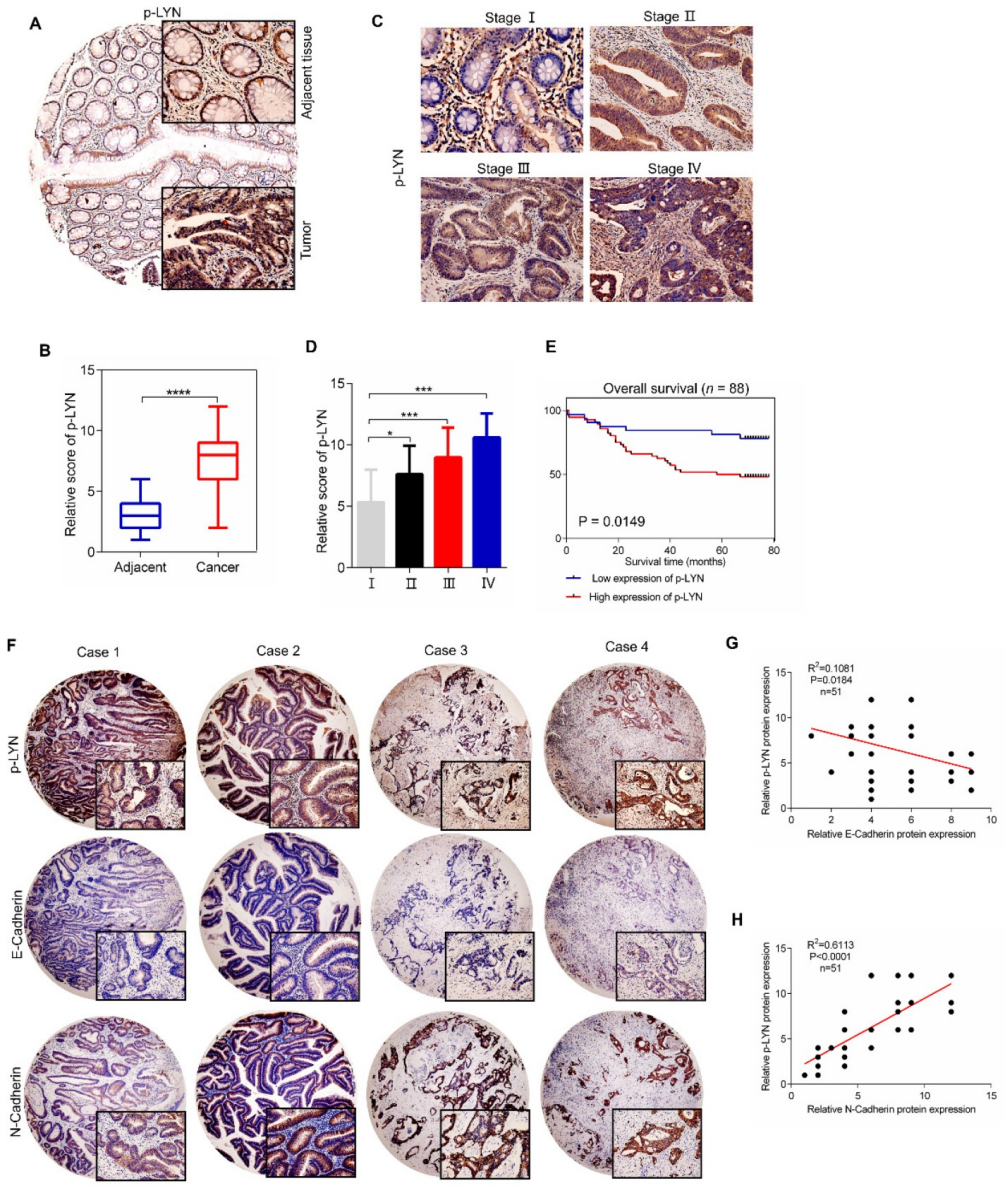
** p-LYN level is elevated in CRC and positively correlates with CRC metastasis. (A)** Immunohistochemical staining was performed to examine the expression of p-LYN in the patient's CRC tissues and adjacent tissues. **(B)** The score of p-LYN staining in (A). **(C)** Immunohistochemical determination of p-LYN expression in each pathological stage of CRC. **(D)** The score of p-LYN staining in (C). **(E)** Kaplan-Meier analysis of the overall survival of 88 CRC patients based on PSAT1 expression. **(F)** Representative images of immunohistochemical staining of p-LYN, E-Cadherin, and N-Cadherin in CRC tissues. **(G-H)** Correlation analysis of the immunostaining intensity of p-LYN with E-Cadherin (G) or N-Cadherin (H). ^**^ P < 0.01; ^***^; P < 0.001; ^****^, P < 0.0001.

We next investigate the clinical relevance of LYN activation in CRC progression using a tissue microarray containing 88 CRC tissues and paired adjacent tissues. Immunohistochemical analysis showed that the level of p-LYN in CRC tissues was increased compared with adjacent mucosal tissues (Figure 6A-B). We also analyzed the correlation between p-LYN level and the clinical stages of CRC, and found that the phosphorylated LYN level was relatively lower in early-stage tumors (stage I~II) and higher in advanced tumors (stage III~IV) (Figure 6C-D). In addition, Kaplan-Meier survival curve showed that the overall survival of CRC patients with high p-LYN expression was significantly shorter than that of patients with low phosphorylated LYN level (Figure 6E). These results indicate that LYN activation is positively correlated with CRC metastasis.

Since we have demonstrated *in vitro* that LYN promotes CRC metastasis by regulating EMT process, we then interrogate the role of LYN activation in EMT in 51 paired human CRC tissues. As shown in Figure 6F-H, the p-LYN level was negatively correlated with E-Cadherin expression, and positively correlated with the expression of N-Cadherin. Collectively, these data suggest that LYN-induced EMT is required for the metastasis of CRC.

### Minocycline inhibits the metastasis of a variety of cancers through LYN-STAT3 signaling

**Figure 7:**
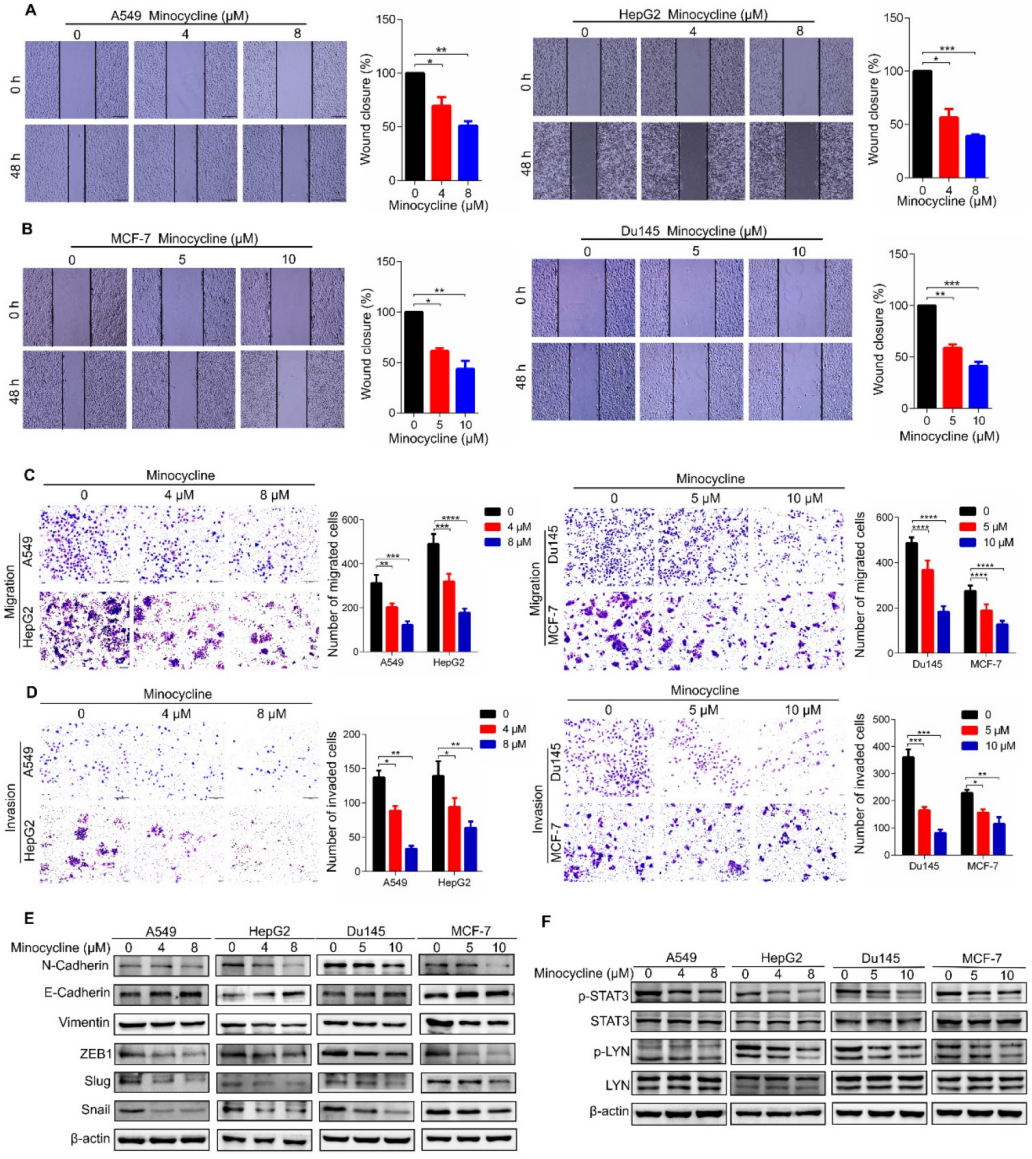
** Minocycline inhibits the metastasis of a variety of cancers through the LYN-STAT3 signaling pathway. (A-B)** Wound-healing migration assays for A549, HepG2, Du145 and MCF-7 cells treated with minocycline. The wound space was photographed at 0 and 48 h. Representative images of wounds and the statistic results were recorded. **(C-D)** Representative images and statistics of Transwell migration and Matrigel invasion assays in A549, HepG2, Du145 and MCF-7 cells treated with minocycline. **(E)** The expression of EMT markers detected by western blot analysis in A549, HepG2, Du145 and MCF-7 cells treated with minocycline. **(F)** The protein levels of LYN, p-LYN, STAT3 and p-STAT3 determined by western blot analysis in A549, HepG2, Du145 and MCF-7 cells treated with minocycline. Error bars indicate the mean ± SD of three independent experiments. ^*^, P < 0.05; ^**^, P < 0.01; ^***^, P < 0.001; ^****^, P < 0.0001.

To investigate the therapeutic effect of minocycline on other cancer types, we first examined the anti-metastatic capacity of minocycline in breast, lung, liver and prostate cancer cells using wound-healing migration assays. The results showed that minocycline significantly inhibited the mobility of breast, lung, liver and prostate cancer cells in a dose-dependent manner (Figure 7A-B), Consistently, minocycline treatment also dose-dependently reduced the migration and invasion of these cancer cells as evidenced by transwell migration and matrigel invasion assays (Figure 7C-D). In addition, minocycline treatment resulted in an increase in the E-Cadherin level and decreases in the levels of N-Cadherin, Vimentin, Slug and Snail in these cancer cells (Figure 7E). The phosphorylation levels of p-LYN and p-STAT3 were found to be inhibited following minocycline treatment in these listed cancer cells (Figure 7F). The above data indicates that minocycline inhibits the metastasis of various cancers by suppressing LYN-STAT3 axis.

## Discussion

Minocycline, a bacteriostatic antibiotic, has been reported to exhibit anti-inflammatory, antioxidant, immune regulation and neuroprotective effects 21. In this study, we found that minocycline inhibits the metastasis of cancer cells, including colorectal, breast, lung, liver and prostate cancer. We identified LYN, a Src family member, as the direct binding target of minocycline. Through direct binding, minocycline inhibits the kinase activity of LYN, leading to reduced STAT3 activation, thereby represses the EMT and metastasis of cancer cells. Our findings reveal an unreported mechanism of minocycline's anticancer effect, and expand the clinical use of minocycline.

Drug repurposing has recently attracted much attention for developing novel drugs for cancer therapy. Repurposing non-oncology drugs for clinical cancer management provides a cost-effective and time-saving strategy for drug discovery 31. To date, many non-oncology drugs have been found to exert promising anticancer effect, such as the antidiabetic drug metformin 32, 33, the sedative thalidomide 34, and the anti-alcohol abuse drug disulfiram 35. In this study, we found that antibiotic minocycline has potent anticancer effect *in vitro* and *in vivo* by inhibiting EMT and metastasis. Notably, a phase II clinical trial study revealed that minocycline treatment has a low-toxicity profile 36.These studies suggest that minocycline has the potential to be repurposed for the treatment of a wide spectrum of cancer types.

Target identification is critical for drug repurposing. Using a chemical proteomics strategy, we found that the Src family kinase LYN is a direct binding target of minocycline. Minocycline binds to the Ala255 of LYN and inhibits the kinase activity of LYN, leading to the suppression of EMT and metastasis of CRC cells. LYN has been reported to be overexpressed in various cancers, including CRC 10-13. Previous studies showed that LYN promoted CRC progression by activating ERK1/2 and AKT-cofilin axis, respectively 37, 38. In this study, we found that minocycline-inhibited LYN represses STAT3 signaling and EMT process, leading to decreased metastasis. This finding is consistent with previous studies that LYN binds with and activates STAT3 to promote the expression of EMT-inducing transcription factors 15, 30, 39.Our study together with previous reports suggest LYN as a therapeutic target for cancer treatment. Of note, our study also highlights minocycline as an optional inhibitor for LYN kinase, the structure of which can be further chemically optimized to obtain better inhibitory affinity. In addition, the anticancer activity of minocycline is not limited to CRC, but also can be recapitulated in other cancer types, indicating a wide-spectrum anticancer efficacy of minocycline.

Our data show that minocycline directly binds and inhibits LYN activity, leading to inhibition of EMT and metastasis of cancer cells by suppressing STAT3 signaling. Our studies unravel the mechanism of action of minocycline's anticancer effect, and suggest minocycline as a promising candidate for drug repurposing in cancer therapy.

## Materials and methods

### Cell culture

SW480 and SW620 (Human colon cancer cell lines), CT26 (Mouse colorectal cancer cell line), HepG2 (Human liver cancer cell line), Du145 (Human prostate cancer cell line), A549 (Human lung cancer cell line) and MCF-7 (Human breast cancer cell line) were purchased from National Collection of Authenticated Cell Cultures (Shanghai, China).293T were purchased from the American Type Culture Collection (ATCC). All cell lines were received as early passages and cultured in DMEM or RPMI-1640 supplemented with 10% fetal bovine serum (FBS) (Biological Industries, Israel). Cells were maintained at 37 °C in humidified atmosphere containing 5% CO_2_. The medium was changed every other day, cells were passaged using 0.25% trypsin (Solarbio, China) and preserved at early passages. All cell lines were free of mycoplasma contamination.

### Reagents

Antibodies for western blotting: LYN (2796), p-LYN (Tyr507) (2731), Vimentin (5741), N-Cadherin (13116), E-Cadherin (3195), Snail (3879), Slug (9585), ZEB1 (3396), STAT3 (4904), p-STAT3 (Y705) (9145) were obtained from Cell Signaling Technology. Flag (390002), GAPDH (200306-7E4), β-actin (200068-L-8F10) were purchased from ZENBIO.

Antibodies for immunohistochemical staining: LYN (2796) and Vimentin (5741) were obtained from Cell Signaling Technology. E-Cadherin (ab76055) was purchased from Abcam. N-Cadherin (22018-1-AP) was purchased from Proteintech. p-LYN (Tyr507) (bs-3256R) was obtained from Bioss.

Minocycline (M102970-5g) was from Shanghai Aladdin Bio-Chem Technology Co. LTD. Epoxy-actived µSphere (P056) was from Pae Biotechnology Co. LTD. Advance transfection reagent (AD600150) was from ZETA.

### Wound-healing migration assay

Wound-healing migration assay was performed in SW480, SW620, A549, HepG2, Du145 and MCF-7 cell lines following treatment with minocycline. Cells were seeded in 6-well plates with a density of 1.0×10^5^/well. When the cells are completely confluent, cell were scraped using a 250 μL sterile pipette, and gently washed 3 times with FBS-free medium to remove the separated cells. Microscopic images of the wounds and migrating cells were obtained at 0, 24 hours and 48 hours. The level of wound closure was measured.

### Transwell migration and matrigel invasion assays

Following the treatment of minocycline, transwell migration and matrigel invasion assays were performed in SW480, SW620, A549, HepG2, Du145 and MCF-7 cell lines. Matrigel (Corning, USA) was thawed on ice and diluted with FBS-free medium. The transwell chamber (Solarbio, China) was treated with 50 μL pre-diluted matrigel and incubated at 37 °C for 2-3 h for solidify. Cells were resuspended in FBS-free medium, 200 μL cell suspension (1×10^5^ cells/mL) was added to the upper compartment and 600 μL medium supplemented with 10% FBS was added into the lower chamber, followed by incubation at 37 °C in 5% CO_2_ for 48 h. Cells remaining on the top of the transwell were then scraped with a cotton swab. The infiltrating tumor cells were washed twice with PBS and fixed with 4% paraformaldehyde for 20 minutes, and then stained with crystal violet.

### Western blotting

Total protein lysates were prepared in RIPA buffer (50 mM Tris, 150mMNaCl, 0.1%SDS, 1%NP-40, pH7.6) with freshly 1% protease inhibitor cocktail (Roche, USA). Protein concentrations were quantified using the Bradford Protein Assay. Proteins were separated by 10% SDS-PAGE gels, transferred onto polyvinyllidene fluoride (PVDF) membranes (Immobilon, China) and blocked with 5% skimmed milk in TBST for 1 h. The membranes were probed with primary antibodies at 4 °C overnight, and then washed with TBST followed by incubation with HRP secondary antibodies (ZENBIO, China). Proteins were detected and visualized using the chemiluminescent HRP substrate (Millipore). Image J software was applied for quantification.

### Plasmids

pCDNA3.1-Flag-LYN plasmid, and pCDNA3.1-LYN (A255D), pCDNA3.1-LYN (M322), pCDNA3.1-LYN (D329A) mutants were constructed by from Shanghai Abs Biotechnology Co., Ltd.

### Expression of recombinant LYN (rLYN)

pET32a-rLYN plasmid was transformed into *E. coli* BL21 (DE3). Isopropyl-β-D-thiogalactoside (IPTG) was used to induce the protein expression of rLYN. The rLYN protein was then purified by gel filtration chromatography (Chromdex 75 PG) and stored at -70 °C for use 40, 41.

### Mass spectrometric analysis

Protein bands visualized via Coomassie Brilliant Blue staining was excised from SDS-PAGE gel and digested in 50 mM ammonium bicarbonate buffer containing RapiGest (Waters Corporation) overnight at 37 °C with 200 ng of modified sequencing-grade trypsin (Promega), and the digested samples were analyzed using high-sensitivity liquid chromatography tandem mass spectrometry with an Orbitrap Fusion Lumos mass spectrometer (Thermo Fisher Scientific) 42, 43.

### Surface plasmon resonance (SPR)

Surface plasmon resonance (SPR) was performed by Biocore T200 biosensor system (GE Healthcare Life Sciences, Piscataway, NJ, USA) at 25℃. According to the formula, the coupling amount of LYN was about 10,000RU. The CM5 chip was separated into 2 channels using the microfluidic chip system (IFC), and each channel was further divided into 2 channels. Channel 1 was used as the reference channel, and channel 2 was coupled to the ligand (rLYN protein). The flow rate was set as 10 μL/min, and the EDC/NHS mixture was flowed through channels 1 and 2 for 60 s. The ligand was diluted to 10μg/mL with pH 5.0 10 mM NaAC solution, and flowed through 2 channels for 90s. Minocycline was serially diluted and flowed through channel 1 and 2 sequentially for 120 s (flow rate 30 μL/min), followed by dissociation for 600 s. The response value was obtained for each concentration. The 1:1 binding model of Bia-evaluation analysis software was used to calculate kinetic parameters.

### LYN enzyme activity assay

The activity of LYN kinase was examined by LYN enzyme activity assay kit (Sigma, CS0730) according to the manufacturer's protocol. Briefly, LYN protein was immunoprecipitated by anti-LYN antibody and EZview^TM^ Red Protein A Affinity Gel beads (P 6486) from the cell lysates. The bead pellets were washed by ice cold 1×wash buffer (W3264) and then suspended in 15 mL of assay buffer(A7354) containing the γ-32P-ATP (W3264) for incubation for 30 minutes at 30 °C. The reactions were terminated by spotting 10 mL of the liquid phase of the assay mixture on 2 cm ×2 cm phosphocellulose P81 squares (P5497). The phosphocellulose squares were then soaked in 0.5% phosphoric acid (79617) and washed once with ethanol and then acetone for 1 minute. The phosphocellulose squares were dried at room temperature or under a heat lamp, followed by detection of the incorporated radioactivity using Cerenkov mode. This experiment was performed at the Department of Radiological Medicine, College of Basic Medicine, Chongqing Medical University (Chongqing, China).

### Animal study

Female BALB/c nude mice (5 weeks old) and BALB/c mice were reared under conditions without specific pathogens. The animal care and experiment protocols conform to the guidelines provided by the Institutional Animal Care and Use Committee of Chengdu Medical College (Chengdu, China). The protocol for the research project has been approved by the Ethics Committee of the Chengdu Medical College. For the experimental mice lung metastasis model, 1×10^6^ SW480 and SW620 cells were injected into nude mice through the tail vein. Minocycline was intraperitoneally injected every other day. Nude mice were killed 6 weeks after injection to examine the lung metastasis of tumor cells. There are two types of lung metastasis models for BALB/c mice. One is that 1× 10^5^ CT26 cells treated with minocycline for 48 hours were injected into the mice through the tail vein, and the mice were killed after 2 weeks to examine the lungs of tumor cells. Another is that 1×10^5^ CT26 cells were injected into mice through the tail vein and treated with minocycline intraperitoneally every other day. After feeding for 2 weeks, the mice were killed to check the lung metastasis of tumor cells. Tumor lung metastasis was confirmed by hematoxylin and eosin staining. Quantification of metastatic nodes was performed based on visual inspection and manual counting.

### Human tissue

Human tissue chip slides were purchased from Shanghai Outdo Biotechnology Company Ltd. They are a set of 180-point survival tissue microarray for colon adenocarcinoma (HColA180Su19), and 4 sets of 60-point tissue microarray for colon cancer (HColA060CD01). The samples come from the National Human Genetic Resources Sharing Service Platform (2005DKA21300). All participants signed informed consent in accordance with the National Human Genetic Resources Sharing Service Platform and fully protected the identity and privacy of all participants. Meanwhile, the protocol for the research project has been approved by the Ethics Committee of Chengdu Medical College and it conforms to the provisions of the Declaration of Helsinki.

### Immunohistochemistry

Human CRC tissue chip slides were purchased from Shanghai Outdo Biotechnology Company Ltd. The tissue chip was dewaxed and rehydratedand then incubated with 0.3% H_2_O_2_ for 30 min to block endogenous peroxidase activity. Antigen retrieval and serum blocking were then performed followed by incubation of primary antibody overnight at 4°C. The slides were washed and then incubated with horseradish peroxidase-labeled secondary antibody for 1 h. Antigen expression was detected by DAB and examined by microscopy. The staining intensity was scored as 0, 1, 2 and 3 for the representation of negative (no staining), mild (weak), intermediate (medium) and intense (strong) staining, respectively. The staining intensity and stained area percentage were multiplied to make a weighted score. The scoring was determined by three independent evaluators.

### Statistical analysis

The values are expressed as mean± SD. Two-tailed Student's t-test, ANOVA, Pearson correlation and log-rank test (GraphPad Software) were used for statistical analysis. The data points were not excluded. *P* < 0.05 was considered significant. Detailed descriptions of statistical tests are specified in the figure legends and in the results.

## Supplementary Material

Supplementary figures.

## Abbreviations

CRC: Colorectal cancer
STAT3: Signal transducer and activator of transcription 3
EMT: Epithelial-mesenchymal transition
