# Supplementary material for: Minocycline binds and inhibits LYN activity to prevent STAT3-meditated metastasis of colorectal cancer
Source: Int J Biol Sci. 2022 Mar 21;18(6):2540–52. doi: 10.7150/ijbs.70708 (PMC8990469; doi:10.7150/ijbs.70708)
Supplement: Supplementary file 1 — Supplementary figures. [file ijbsv18p2540s1.pdf]

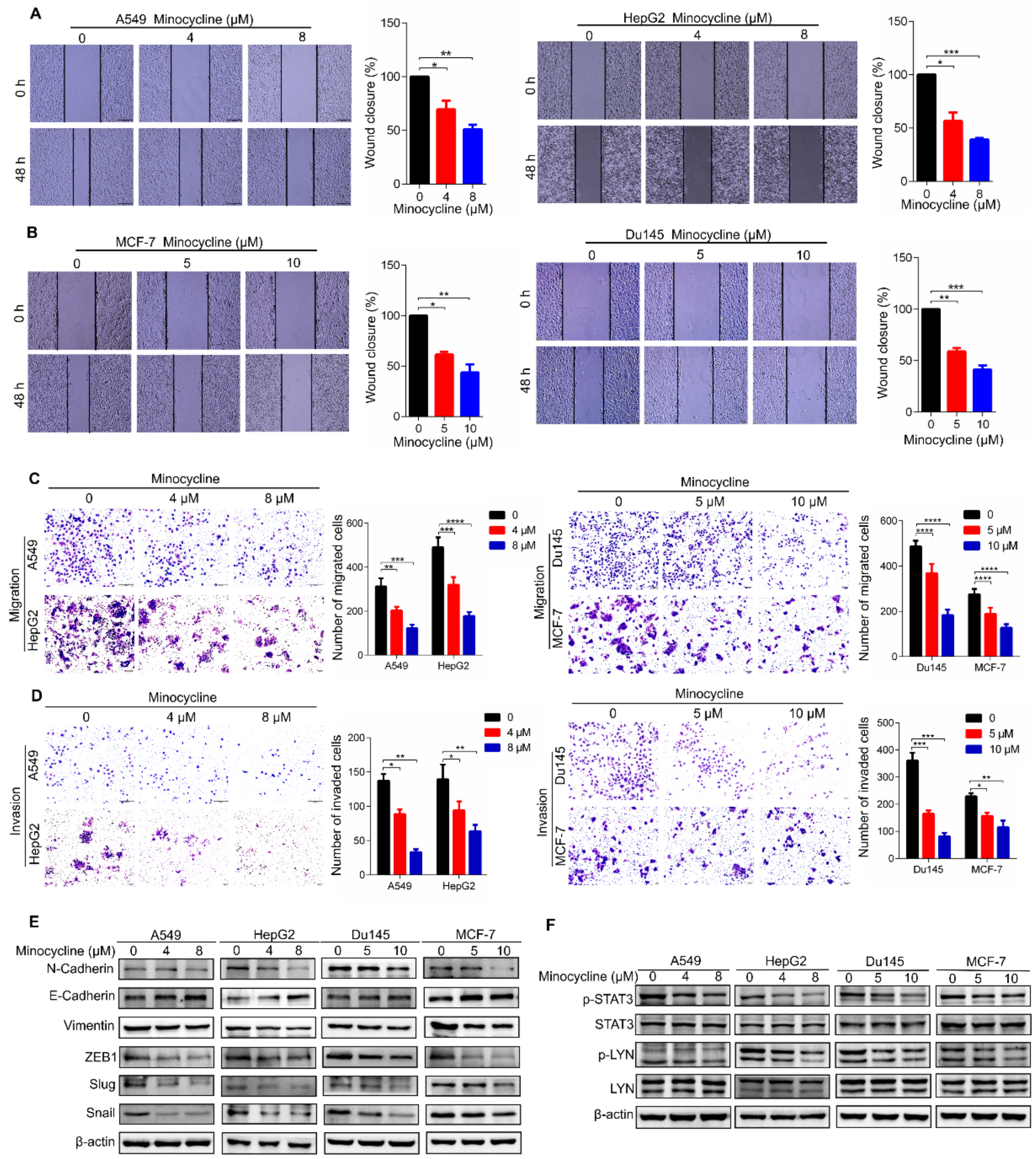

Supplementary Figure 1. Minocycline has no obvious effect on the CRC cell growth at relatively low concentrations. (A-B) CRC cell lines SW480 and SW620 were treated with various concentrations of minocycline for 48h and subjected to cell viability assay. (C) Colony formation assay of SW480 and SW620 cells treated with the indicated concentrations of minocycline. Representative images (Left) and quantification of colonies (Right) were shown. Error bars indicate the mean ± SD of three independent experiments. **, P < 0.01; ***, P < 0.001; ****, P < 0.0001.

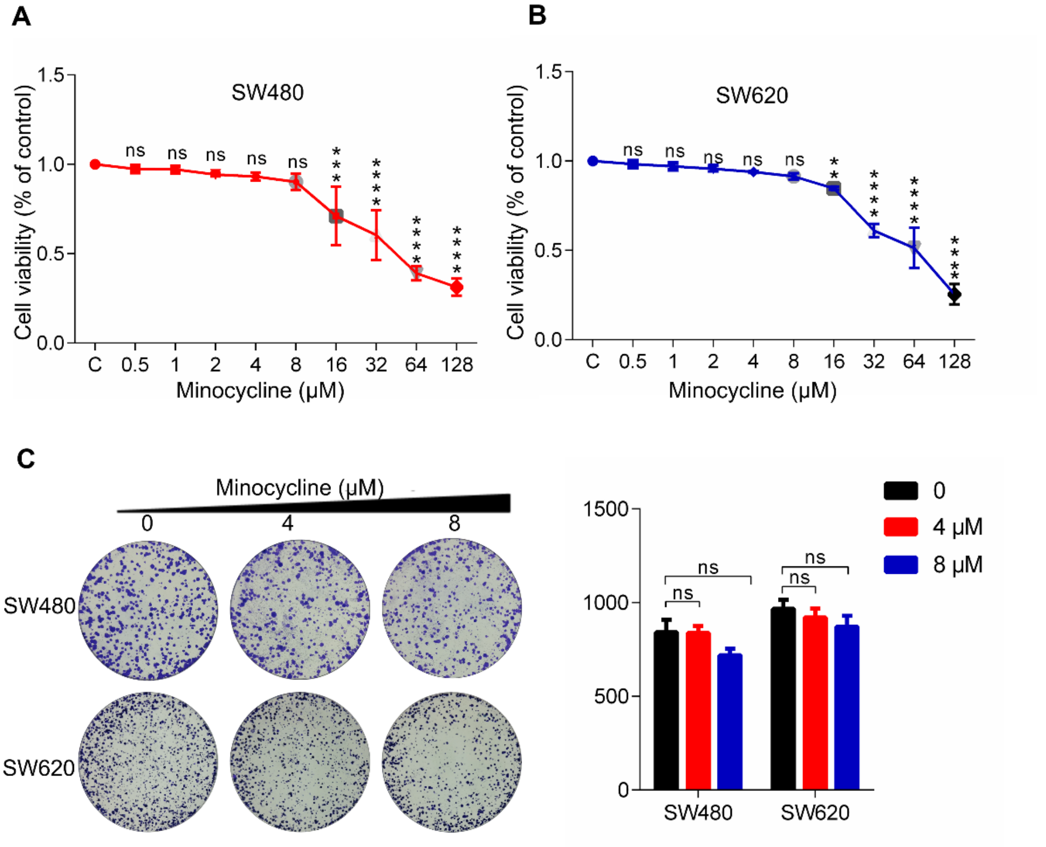

Supplementary Figure 2. Minocycline inhibits the lung metastasis. (A) Representative images and statistical analysis of the number of lung metastatic nodules following injection of minocycline-treated CT26 cells. (B-C) Representative images of H&E staining (B) and the weight of mice lung (C) following injection of minocycline-treated CT26 cells. *, $P < 0.05$; **, $P < 0.01$; ***, $P < 0.001$.
